# Effects of intramammary infusion of sage (*Salvia officinalis*) essential oil on milk somatic cell count, milk composition parameters and selected hematology and serum biochemical parameters in Awassi sheep with subclinical mastitis

**DOI:** 10.14202/vetworld.2017.895-900

**Published:** 2017-08-10

**Authors:** Myassar O. Alekish, Zuhair B. Ismail, Mofleh S. Awawdeh, Shoroq Shatnawi

**Affiliations:** 1 Department of Veterinary Clinical Sciences, Faculty of Veterinary Medicine, Jordan University of Science and Technology, Irbid, Jordan; 2 Department of Veterinary Pathology and Public Health, Faculty of Veterinary Medicine, Jordan University of Science and Technology, Irbid, Jordan; 3 Private Large Animals Practice, Bushra, Jordan

**Keywords:** antibiotics, alternative treatment, Awassi sheep, mastitis

## Abstract

**Aim:**

The aims of this study were to evaluate the effects of intramammary infusion of sage (*Salvia officinalis*) essential oil (EO) on milk somatic cell count (SCC), milk composition parameters and selected hematology and serum biochemical parameters in 20 Awassi ewes affected with subclinical mastitis.

**Materials and Methods:**

The dried leaves of sage were used to extract the EO by hydrodistillation. The minimum inhibitory concentration (MIC) and minimum bactericidal concentration (MBC) of sage EO against *Staphylococcus aureus* were determined by the broth dilution method. Ewes were divided randomly into three main groups and received one of the following treatments; Group 1 (n=5): Dimethyl sulfoxide (DMSO) alone (5 ml; 0.2 ml of DMSO in 4.8 ml of saline), Group 2 (n=5): Amoxicillin alone (3 ml), and Group 3 (n=10): Sage EO (5 ml of sage EO solution [0.2 ml DMSO+1 ml EO+3.8 ml sterile saline]). All treatments were administered by intramammary infusion into each teat twice per day for 3 consecutive days. Milk samples for SCC and milk components determination and whole blood samples for hematology and serum biochemical analyses were collected before treatment (T0) and at 24 (T24) and 48 (T48) h after the last treatment.

**Results:**

The MIC and MBC of sage EO against *S. aureus* were 12.5% and 6.1%, respectively. SCC was decreased significantly (p<0.05) at T24 and T48 h in sage EO and amoxicillin treated groups. Milk fat and lactose were increased significantly (p<0.05) in sage EO and amoxicillin treated ewes while no significant changes were observed in the percentages of solids-not-fat, protein and total solids. No significant effects of sage EO treatment on any of the hematology or serum biochemical parameters were observed. There were no local or systemic side effects observed in any of the treated ewes. However, further clinical trials are warranted to determine safety and possible withdrawal times in milk before its recommendation for use in organic operations.

**Conclusion:**

In this study, the intramammary infusion of sage EO to ewes affected with subclinical mastitis resulted in a significant decrease in SCC 24 h and 48 h posttreatment. In addition, milk fat and lactose were increased in animals that received the EO as well as in those treated with the antibiotic.

## Introduction

Mastitis is a common and costly disease affecting all milk producing animals [1]. The disease has important economic and animal welfare impacts. Economic losses are mainly due to costs of veterinary care and drugs, loss of production, death or culling of animals and reduced milk price due to poor quality [2]. Mastitis, whether it is clinical or subclinical is known to negatively affect udder tissues, productivity and milk constituents such as casein, lactose, and fat further contributing to the economic burden on farmers [3]. Mastitis is also known to seriously impact the general health and well-being of animals causing significant animal welfare concerns impacting consumer and public perspectives.

Antibacterial agents have been the mainstay of mastitis treatment and prevention programs for decades. The use of antibacterial agents has been accompanied by the appearance of resistant strains of common bacterial species in dairy animals [4]. This rising concern has led to the urgency of finding new and innovative treatment options for mastitis worldwide. In the past few decades, a large quantity of research has been focused on characterizing the antibacterial effects of different herbs and aromatic plants and many other natural substances for the treatment of different animal diseases including mastitis [5,6].

Sage (*Salvia officinalis*) is a common herb that can grow wildly or in cultivation. It has been long known for its broad medicinal properties including anti-inflammatory, antiseptic, and antibacterial effects. The active component of sage essential oil (EO) is 1, 8-cineole which was particularly found affective against *Escherichia coli* and *Staphylococcus aureus* [7,8]. The objectives of this study were to evaluate the effects of intramammary infusion of sage EO on milk somatic cell count (SCC), various milk component parameters and selected hematology and serum biochemical parameters in Awassi sheep affected with subclinical mastitis.

## Materials and Methods

### Ethical approval

This to declare that the Animal Care and Use Committee (ACUC) at Jordan University of Science and Technology has approved that all adequate measures were taken to minimize pain or discomfort to all the animals included in this study. The experiment was carried out in accordance with the Guidelines laid down by the International Animal Ethics Committee and in accordance with local laws and regulations.

### Sage EO extraction

The EO of sage was extracted by hydrodistillation using Clevenger’s apparatus. Briefly, freshly collected sage plants from northern Jordan were left to air dry. The leaves were then weighed and mixed with distilled water at a rate of 1 g sage leaves per 6.81 ml of distilled water. The mixture was then heated to 100°C for 3 h. The EO was then collected. The extracted oil was dehydrated by adding a few drops of sodium sulfate (0.5 g for 4 ml EO) and stored at −20°C until used.

### Minimum inhibitory concentration (MIC) and minimum bactericidal concentration (MBC)

The MIC of sage EO was performed against previously isolated pathogenic strains of *S. aureus* from field cases of mastitis. MIC was determined by the broth dilution method in Muller-Hinton broth. Briefly, sage EO was first dissolved in 80% (v/v) dimethyl sulfoxide (DMSO) solution. As DMSO is considered to have an antibacterial effect so this study tested different concentrations to avoid the effect of DMSO showed the least effect. To determine the best DMSO dilution, different concentrations were made and smeared on an already streaked bacterial culture plates using *S. aureus* field strain and incubated at 37°C for 24 h. DMSO concentration (0.2 ml of DMSO plus 4.8 ml of distilled water) that induced least bacterial inhibition was chosen. This was called the stock solution or solution I. Different concentrations of sage EO were then obtained using distilled water as follows: 100% (Solution I), 50%, 25%, 12.5%, 6.2%, and 3.1% (v/v), all called solution II.

To determine the MIC, 1 ml of *S. aureus* inoculum (10^6^ UFC/ml) and 1 ml of each EO dilutions (Solution II) were added to 2 ml of Muller-Hinton broth. Positive control was prepared by adding 1 ml of *S. aureus* inoculum to 3 ml of Muller-Hinton broth. Negative control was prepared by adding 1 ml of *S. aureus* inoculum to 1 ml of DMSO and 2 ml of Muller-Hinton broth. After 24 h of incubation at 37°C, MIC was determined as the lowest concentration of the EO inhibiting visible bacterial growth.

To determine the minimum bactericidal concentration, 10 µl of bacterial inoculum was taken aseptically from tubes that had not presented visible turbidity and was spread on blood agar plates and incubated at 37°C for 24 h [9]. The MBC was considered as the lowest concentration of EO that allowed <0.1% of the original inoculum treated with the EO to grow on the surface of the blood agar.

### Dosage calculations and preparation

The effective dose was considered 10 times the MIC [10,11]. Dosage was, therefore, prepared by adding 0.2 ml of DMSO to 1 ml of 12.5% sage EO solution. The total volume was then made to 5 ml by mixing with 3.8 ml of sterile saline. The prepared dose was then injected into each teat at 12 h intervals after milking, for 3 consecutive days following the appropriate aseptic preparation of the teat.

### Animals and study design

A total of 20 lactating ewes aged between 2 and 5 years, located in Al-Mafraq Governments of Jordan were used in this study. Before the enrollment in the study, all ewes were subjected to a complete physical examination including udder palpation. Milk samples were then collected from each ewe, and California mastitis test (CMT) was performed.

Only ewes are suffering from subclinical mastitis based on a positive CMT and SCC more than 250,000 cells/ml were included in the study. Enrolled ewes were divided randomly into three main groups and received one of the following treatments; Group 1 (n=5): DMSO alone, Group 2 (n=5): Amoxicillin alone, and Group 3 (n=10): Sage EO. In the DMSO group, each ewe received 5 ml of DMSO (0.2 ml of DMSO in 4.8 ml of saline) in each udder at 12 h intervals for 3 consecutive days. In the amoxicillin group, each ewe received 3 ml tube of an intramammary preparation (Synulox, Pfizer Animal Health, UK) in each udder half at 12 h intervals for 3 consecutive days. In Groups 3, sage EO solution (0.2 ml DMSO+1 ml EO+3.8 ml sterile saline) was injected into each udder half at 12 h intervals for 3 consecutive days.

All ewes were closely monitored after infusion for immediate adverse reactions and were kept under observation for the next 5 days.

### Milk and blood samples collection

Milk samples were collected from all ewes with positive CMT and placed in test tubes for SCC and milk constituent analyses. Milk samples were collected before the treatment (T0) and at 24 (T24) and 48 (T48) h after the last EO administration. Milk samples were collected according to the standard procedures of International Dairy Federation [12]. In addition, whole blood samples were collected before the treatment (T0) and at 48 h after the last EO administration. Blood was collected via jugular vein puncture and placed in plain test tubes and EDTA-containing tubes for serum biochemistry and hematology analyses, respectively. All samples were transported to the laboratory in ice box and tested within 2 h.

### SCC

SCC was determined by spreading 10 µl of milk on 1 cm^2^ area of glass slide. Slides were left to air dry before they were stained using Newman-lambert stain. Stained slides were examined under light microscopy, and somatic cells were counted manually as described previously [13].

### Milk constituents

Milk composition parameters including fat, protein, solids, solid-not-fat (SNF) and lactose were measured automatically (Milko scope julie c8; Scope Electric, Regensburg, Germany) according to the manufacturer’s instructions.

### Hematology and serum biochemical analyses

Whole blood samples were collected from the jugular vein and placed in plain test tubes and EDTA-containing tubes before the experiment (T0) and 48 h (T48) after the last EO administration. Samples were transported in ice box to the laboratory for hematology and blood chemistry. Hematology analysis included the determination of white blood cells, red blood cells, hemoglobin concentration, packed cell volume (PCV), mean corpuscular hemoglobin, fibrinogen, and platelet count using ABC Vet hematology analyzer (ABX Diagnostics, France). For serum biochemical parameters, blood urea nitrogen (BUN), creatinine, aspartate aminotransferase, and alanine aminotransferase were determined using commercially available kits.

### Statistical analysis

Data are presented in means±standard error. All data were analyzed statistically using the Mixed procedure of SAS System for Windows Release 8.1 (2002, SAS Inst. Inc., Cary, NC) as repeated measures according to a completely randomized design. The model contained the effects of treatment, time, and interactions between treatment and time. Treatment means were computed using the LSMEANS option and separated using preplanned pairwise comparisons of least squares means using t-tests. Statistical difference was considered significant at p<0.05.

## Results

### MIC and MBC of sage EO

The MIC of sage EO against *S. aureus* was determined to be 12.5% which equals to 120 mg EO while the MBC was determined to be 6.1%.

### Effects of sage EO on SCC

SCC was decreased significantly at 24 and 48 h after treatment using both sage EO and amoxicillin (Table 1). In the sage EO group, SCC was 2000×10^3^ cells/ml at 48 h after the last treatment while it was 3400×10^3^ cells/ml before the treatment. In ewes treated with amoxicillin, SCC was 1500×10^3^ cells/ml 48 h after treatment compared to 2900×10^3^ cells/ml before the treatment. In the DMSO treated group, SCC actually increased slightly after administration.

**Table-1:** Effects of intramammary infusion of sage EO on milk somatic cell count (×10^3^cells/ml) in Awassi sheep affected with subclinical mastitis (mean±SD).

Groups	Time of sampling		

	T0	T24	T48
Sage EO	3400±570^1^	1900±330^2^	2000±460^2^
DMSO	2600±150	2900±567	2800±345
Amoxicillin	2900±350^1^	2000±547^2^	1500±590^2^

T0=Before treatment, T24=24 h after treatment, T48=48 h after treatment, EO=Essential oil, SD=Standard deviation, DMSO=Dimethyl sulfoxide. Different superscript numbers in a row are significant at p<0.05

### Effects of sage EO on milk constituents

Milk fat and lactose were increased significantly in sage EO and amoxicillin treated ewes (Table 2). In sage EO and amoxicillin treated groups, fat percentages increased from 4.6 to 6.7 and from 5.9 to 7.5, respectively. While in DMSO, fat percentage did not change significantly.

**Table-2:** Effects of intramammary infusion of sage EO on various milk constituents (%) in Awassi sheep affected with subclinical mastitis (mean±SD).

Group	Sage EO		Amoxicillin		DMSO	
		
	T0	T48	T0	T48	T0	T48
Fat	4.60±0.80	6.70±0.80*	5.90±1.0850	7.50±1.00*	4.50±1.00	6.10±1.00
SNF	8.70±0.17	9.40±0.18	10.00±0.60	11.00±0.60	8.80±0.60	9.60±0.60
Protein	5.00±0.40	4.10±0.40	5.20±0.40	5.80±0.40	5.10±0.40	5.00±0.40
Lactose	3.80±0.30	4.50±0.30*	3.30±0.20	4.40±0.20*	3.80±0.20	3.80±0.20
Solids	0.80±0.04	0.75±0.04	0.80±0.05	0.90±0.050	0.80±0.05	0.80±0.05

SNF=Solids-not-fat, EO=Essential oil, SD=Standard deviation, DMSO=Dimethyl sulfoxide.

* p<0.05 using repeated measures

In sage EO and amoxicillin treated groups, lactose percentages also increased significantly after treatment. Before treatment, lactose percentages were 3.8 and 3.3 in sage EO and amoxicillin treated groups, respectively. After treatment, lactose percentages increased to 4.5 and 4.4 in sage EO and amoxicillin treated groups, respectively.

Milk SNF percentages were not significantly increased in both sage EO and amoxicillin treated ewes. There were no significant changes in the percentages of SNF in DMSO treated groups.

SNF percentages increased from 8.7 to 9.4 and from 10 to 11 in sage EO and amoxicillin treated groups, respectively. No significant changes in the percentages of SNF in DMSO treated groups.

There were no significant effects on the percentages of protein and solids in milk of any of the treated groups.

### Effects of sage EO on hematology and serum biochemical parameters

There were no statistically significant effects of sage EO treatment on any of the hematology or serum biochemical parameters tested in this study (Table 3).

**Table-3:** Effects of intramammary infusion of sage EO on selected blood hematology and serum biochemical parameters in Awassi sheep affected with subclinical mastitis (mean±SD).

Groups	Sage EO		Amoxicillin		DMSO	
		
	T0	T48	T0	T48	T0	T48
PCV (%)	24±0.70	21±0.70	26±0.90	24±0.90	27±0.90	22±0.90
Hb (g/dl)	8.70±0.30	8.80±0.30	9±0.40	11±0.40	9±0.40	9±0.40
WBC (×10^3^/µl)	7.70±0.50	9.70±0.5	7.70±0.80	9.90±0.80	7.6±0.70	7.60±0.80
Urea (mmol/L)	4.80±0.50	6.30±0.60	6±0.80	7±0.80	6±0.80	5.50±0.80
Creatinine (mmol/L)	113±18	87±19	173±25	143±25	103±25	115±25
AST (U/L)	56±4.80	62±5.00	63±6.90	46±6.80	56±6.80	62±6.80
ALT (U/L)	28±3	23±3	29±4	33±4	30±4	22.60±4

PCV=Packed cell volume, Hb=Hemoglobin, WBC=White blood cells, AST=Aspartate aminotransferase, ALT=Alanine aminotransferase, EO=Essential oil, SD=Standard deviation, DMSO=Dimethyl sulfoxide

### Side effects

All sheep tolerated the intramammary infusion of sage EO very well. There were no local or systemic local effects noticed in any of the treated ewes. However, the effect sage EO on milk culinary characteristics and milk withdrawal times were not determined in this study.

## Discussion

The EO of sage has proven *in vitro* antibacterial effects against certain important bacteria causing mastitis in ruminants such as *E. coli* and *S. aureus* [7]. The mechanism of this antibacterial effect was suggested to be due to the active component in Sage 1, 8-cineole [7]. The active component 1, 8-cineole in sage is believed to exert strong hydrophobicity effect on microbial cell wall, and mitochondria leading to leakage of cell contents and ultimately cell death [8]. Although among many other medicinal plants, sage has been used for a long time as an alternative to synthetic drugs for many purposes such as anti-inflammatory, antiseptic, and antibacterial effects, still more scientific evidence and more research to evaluate *in vivo* antimicrobial activity of EOs are needed [14].

SCC is used to identify mastitis in ruminants and can be used to evaluate the effectiveness of various drug regimens including sage EO [15]. The normal range of SCC for sheep milk has been determined previously and is known to vary between 10×10^3^ and 200×10^3^ cells/ml [16]. In this study, SCC in sage EO and amoxicillin treated groups were significantly decreased indicating potential therapeutic effects of this plant against ovine mastitis. Review of recent literature concerning *in vivo* and *in vitro* effects of sage EO against various bacterial species indicates variable results. In one study, weak inhibitory zone of sage EO was reported which indicates poor antibacterial effect [17]. These results were further fortified later by other researchers who concluded that sage EO did not show any antimicrobial activity at the studied concentration [11].

On the other hand, other recent studies have proven potential antioxidant and anti-inflammatory activities of sage EO. In addition, sage EO was found to exert antimicrobial activity against *Staphylococcus* spp., and other microorganisms as reported by Baranauskiene *et al*. [18] and Viuda-Martos *et al*. [19]. Variation in the results of different studies could be explained by variation in extraction techniques (alcoholic method vs. hydrodistillation method) and variation in the dilution methods and concentration of the active components of the EO [11].

Moreover, the effect of sage EO *in vivo* could be significantly different than that reported *in vitro*. This difference could be related to the pH in the media where the oil is supposed to exert its effects. For example, the pH of milk varies between 6.4 and 6.6, but in the case of an infection, it increases to pH of 7.4 [20]. For best results, lower pH such (5-6) is preferred for sage EO [8,21].

Milk composition is an important technological and quality feature of the processed and raw products. Several, animal related such as breed, age, mammary gland health, lactation stage, and monumental factors such as nutrition and dietary composition are responsible for the variation in various milk composition concentrations. Furthermore, both clinical and subclinical mastitis have been associated with a significant effect on milk physical as well as chemical characteristics [12,22]. This scientific data regarding the effect of mastitis on various milk components is variable, however. In general, mastitis may result in an increase in the level of proteinous compounds associated with the inflammatory and immune response such as albumin and immunoglobulins and a decrease in the endogenous milk proteins such as caseins [23,24]. There is also a decrease in the level of lactose and fat due to impaired synthesis [23]. In this study, the percentages of milk fat and lactose increased significantly after treatment of the udder using sage EO indicating a possible improvement in the udder health and milk quality. This was also noticed in ewes treated using a conventional antibiotic treatment which further indicates the effectiveness of this alternative therapy against ovine mastitis.

## Conclusion

The intramammary administration of sage EO to ewes affected with subclinical mastitis in this study resulted in a significant decrease in SCC 24 h and 48 h post administration. In addition, milk fat and lactose were increased in animals that received the EO as well as in those treated with the antibiotic. Further, clinical trials are warranted to determine safety and possible withdrawal times in milk before its recommendation for use in organic operations.

## Authors’ Contributions

MOA: design the experiment, organizing the experimental work; samples collection and laboratory work. Data interpretation and paper writing. ZBI: Data analysis and interpretation. Editing the paper. MSA: Statistical analysis, help in reading and interpreting the data. Paper editing. SS: samples collection and the perform the lab work. help in paper drafting. All authors read and approved the final manuscript.
